# Association between job exposures and employment status 2 years after medical rehabilitation

**DOI:** 10.1093/eurpub/ckac131.274

**Published:** 2022-10-25

**Authors:** M Brünger, K Spyra, S Bernert

**Affiliations:** Institute of Medical Sociology and Rehabilitation Science, Charité - Universitätsmedizin Berlin, Berlin, Germany

## Abstract

**Background:**

Research shows an important association between job exposures and various health- and employment-related outcomes. On contrast, little is known about the impact of job exposures on the employment status after medical rehabilitation. Thus, the aim of this study is to investigate the association between job exposures and employment, unemployment, and disability pension entry 2 years after rehabilitation.

**Methods:**

A retrospective cohort study was performed based on the scientific use file “SUFRSDLV15B” of the German Pension Insurance containing rehabilitation and occupational data at a monthly level. We included n = 597,021 insured persons aged 18 to 63 years completing a medical rehabilitation between 2008 and 2013 in Germany and traced their employment status over a 24-month follow-up period. Job exposures were operationalised with the Overall Job Exposure Index (Kroll, 2015) by applying job-exposure-matrices.

**Results:**

Persons with high job exposures in comparison to those with low job exposures were less likely to be employed (87.6% vs. 92.6%) and more likely to be unemployed (13.9% vs. 7.7%) and to draw disability pension (4.8% vs. 4.4%) for at least one month in the 2-year-period after rehabilitation. One minus survival curves showed that the differences were already evident in the first month after rehabilitation and further increased during the following 24 months. Cox regressions revealed that these associations remained stable when adjusting for gender, age, and employment status before rehabilitation.

**Conclusions:**

The results underline the importance of addressing job exposures during rehabilitation to enhance return-to-work and stay-at-work after rehabilitation. These findings could help to identify particularly vulnerable groups of insured persons based on routine data at an earlier stage than has been the case so far and to give them access to structured workplace-oriented medical rehabilitation programmes that have been established in recent years.

**Key messages:**

• High job exposures are associated with less work participation after medical rehabilitation.

• To increase return-to-work, it may be useful to address job exposures in rehabilitation more than before.

